# Cross-protection between attenuated *Plasmodium berghei* and *P. yoelii* sporozoites

**DOI:** 10.1111/j.1365-3024.2007.00976.x

**Published:** 2007-11

**Authors:** M SEDEGAH, W W WEISS, S L HOFFMAN

**Affiliations:** 1 Malaria Program, Naval Medical Research CenterSilver Spring, MD, USA

**Keywords:** *CD8^+^ T cells*, *cross-protection*, P. berghei, P. yoelii, *sporozoites*

## Abstract

*An attenuated*Plasmodium falciparum *sporozoite (*Pf*SPZ) vaccine is under development, in part, based on studies in mice with*P. berghei*. We used*P. berghei *and*P. yoelii *to study vaccine-induced protection against challenge with a species of parasite different from the immunizing parasite in BALB/c mice. One-hundred percent of mice were protected against homologous challenge. Seventy-nine percent immunized with attenuated*P. berghei *sporozoite (*Pb*SPZ)*(six experiments) *were protected against challenge with*P. yoelii *sporozoite (*Py*SPZ), and 63% immunized with attenuated*Py*SPZ*(three experiments) *were protected against challenge with*Pb*SPZ. Antibodies in sera of immunized mice only recognized homologous sporozoites and could not have mediated protection against heterologous challenge. Immunization with attenuated*Py*SPZ or*Pb*SPZ induced CD8^+^ T cell-dependent protection against heterologous challenge. Immunization with attenuated*Py*SPZ induced CD8^+^ T cell-dependent protection against homologous challenge. However, homologous protection induced by attenuated*Pb*SPZ was not dependent on CD8^+^ or CD4^+^ T cells, and depletion of both populations only reduced protection by 36%. Immunization of C57BL/10 mice with*Pb*SPZ induced CD8^+^ T cell-dependent protection against*P. berghei*, but no protection against*P. yoelii*. The cross-protection data in BALB/c mice support testing a human vaccine based on attenuated*Pf*SPZ for its efficacy against*P. vivax.

## INTRODUCTION

In 1967 it was reported that immunization of A/J mice with radiation-attenuated *Plasmodium berghei*sporozoites (*Pb*SPZ) protected the mice against malaria (1). Several years later, it was reported that humans immunized by exposure to radiation-attenuated *P. falciparum* sporozoites (*Pf*SPZ) (2–4) were also protected. There now is a major effort to develop an attenuated sporozoite (SPZ) vaccine to protect humans against *P. falciparum*, the malaria parasite responsible for greater than 98% of the 1–3 million deaths caused by malaria annually (5). It is not known if immunization with attenuated *Pf*SPZ will protect against exposure to *P. vivax, P. malariae*or *P. ovale*, the three other *Plasmodium* species that infect humans. One individual was immunized with *Pf*SPZ, shown to be protected against *P. falciparum*, and then challenged with *P. vivax* SPZs, and shown not to be protected (6), raising the possibility that there may not be cross-protection. Determination of cross-protection in humans must await clinical trials. However, because of the importance murine studies have played in providing the foundation for human studies with attenuated SPZs, we have used two rodent malaria parasites, *P. berghei*and *P. yoelii*, to study cross-protection in mice. The results indicate that there is significant cross-protection, and that different immune responses may be active against closely related parasite species.

## MATERIALS AND METHODS

### Mice

Female, 6- to 8-week-old BALB/CByJ and C57B1/l0 mice from Jackson Laboratories (Bar Harbor, ME, USA) were used in this study.

### Parasites and immunizations

Sporozoites of *P. berghei*(ANKA) (7) and *P. yoelii*(17NL, 1.l clone) (8) were produced in laboratory-bred and infected *Anopheles stephensi*mosquitoes. Sporozoites for immunization were separated from infected mosquitoes using a renograffin discontinuous gradient as previously described (7). Sporozoites were prepared from infected mosquitoes that had been exposed to 10 000 rad from a ^137^Ce source. Harvested SPZs were counted, diluted to the required concentration in M199 containing 5% normal mouse serum, and a volume of 0·2 mL was injected i.v. through the tail vein. Mice received multiple doses at 2-week intervals consisting of 7 × 10^4^ irradiated SPZs as the first dose and 3 × 10^4^ irradiated SPZs as subsequent doses. In challenge experiments, hand-dissected, infected mosquito glands were triturated in medium to obtain SPZs. Challenge doses varied from 1 to 10 × 10^3^ nonirradiated SPZs and were injected i.v. Minimum infective doses and the 50% infectious dose (ID_50_) of SPZs used to challenge the immune animals were determined during most challenges by making fivefold dilutions of the challenge inocula.

### Antibody responses

An indirect fluorescent antibody test (IFAT) was used to detect antibodies to *P. yoelii* and *P. berghei* SPZs in sera of mice by methods previously described (8). Briefly in the IFAT, diluted sera were reacted with air-dried SPZs and end point titres were detected using fluorescein isothiocyanate (FITC)-labelled rabbit anti-mouse immunoglobulin.

### Protection against challenge

Two weeks after the last immunization, mice were challenged by i.v. inoculation in the tail vein with viable *P. yoelii* or *P. berghei* SPZs. Giemsa stained blood films were examined with a light microscope under an oil immersion objective (1000×) for the presence of asexual erythrocytic stage parasites. Mice showing no parasites in their blood for 15 days following challenge with infective SPZs were designated as negative.

### Depletion of CD8^+^ T lymphocytes

To determine if CD8^+^ T cells were required for protective immunity, we depleted mice of CD8^+^ T lymphocytes just prior to challenge. Mice immunized with five doses of irradiated SPZs were given three 0·5-mg i.p. injections of the mAb 19/178 at 24-h intervals to deplete them of CD8^+^ T lymphocytes as previously described (9). Peripheral lymphocyte samples were prepared and analysed for depletion of the population carrying the CD8^+^ T cell marker in a fluorescent-activated cell sorter (FACS). The mice were challenged with infective SPZs 4 days later. Treatment with mAB 19/178 was continued every third day after challenge until patency occurred. Control immunized mice were injected with the same quantities of rat immunoglobulin.

### Calculation of the ID_50_ and challenge units

Because the infectivity of individual preparations of SPZs may vary, the infectivity of the SPZs used for challenge was monitored during challenge in some of the experiments (Tables 1–3). Serial dilutions of SPZs were inoculated into groups of naive mice to determine the ID_50_ (the number of SPZs needed to infect 50% of injected normal mice) of the SPZs used in the challenge. For *Py*SPZ, 200, 40, 8 or 1·6 SPZs were injected i.v. into groups of six mice. For *Pb*SPZ, 1000, 200 or 40 SPZs were injected i.v. into groups of six mice as *Py*SPZs are more infectious than *Pb*SPZ. To obtain the ID_50_, we took the natural logarithm (ln) of the doses tested. We used logistic regression to model the log odds of infection vs. the log-transformed doses. The ID_50_ is the log dose at which the probability of infection is 0·5. To be acceptable for calculation of the ID_50_, at least two consecutive dilutions had to have less than 100% and greater than 0 infectivity. This is because the log of the odds for these results had to be finite. Confidence intervals for this point estimate were calculated using the asymptotic variance obtained by the delta method; log ID_50_ values were exponentiated back to numbers of SPZs. After determining the ID_50_for the individual SPZ preparation, the number of ID_50_units contained in the challenge dose was calculated by dividing the challenge dose by the calculated ID_50_.

**Table 1:** Cross-protection between *Py*SPZ and *Pb*SPZ in BALB/c mice

		Number protected/ number challenged		
Immunization	Challenge SPZs^a^	Experiment 1	Experiment 2	Total (% protected)
Irradiated *Py*SPZ	*P. yoelii*	6/6	7/7	13/13 (100)
Irradiated *Py*SPZ	*P. berghei*	4/6	3/7	7/13 (54)
Irradiated *Pb*SPZ	*P. berghei*	6/6	7/7	13/13 (100)
Irradiated *Pb*SPZ	*P. yoelii*	5/6	7/7	12/13 (92)
Naive	*P. yoelii*	0/6	0/7	0/13 (0)
Naive	*P. berghei*	0/6	0/7	0/13 (0)

Mice were immunized i.v. with five doses of radiation-attenuated *Py*SPZ or *Pb*SPZ. Two weeks after the last immunization, mice were challenged with infective SPZ. The number of *Py*SPZ used for the challenge was 5 × 10^3^, and that for *Pb*SPZ was 10 × 10^3^. The units of ID_50_ were calculated by dividing the challenge dose by the ID_50_.

a Although we did SPZ titrations to determine the infectivity of the SPZs used in Experiments 1 and 2, we only obtained enough data points (see Materials and methods) in one experiment for each parasite to calculate the ID_50_. The ID_50_ for *P. yoelii* in Experiment 1 was 3·3 SPZs (95% CI 1·2−8·8), and the number of *P. yoelii* ID_50_ units contained in the 5 × 10^3^ SPZs used for this challenge was 1525·7 (95% CI 569·1−4090·3). The ID_50_ for *P. berghei*in Experiment 2 was 370 SPZs (95% CI 126·5–1082·1), and the number of *P. berghei* ID_50_ units contained in the 10 × 10^3^ SPZs used for this challenge was 27·0 (95% CI 9·2–79·1).

## RESULTS

### Antibody responses

Table 4 shows that immunization with irradiated *Py*SPZ or *Pb*SPZ resulted in the production of high antibody titres to the parasite used for the immunization, but no cross-reacting antibodies to the other species.

**Table 4:** Antibodies in BALB/c mice to *Py*SPZ and *Pb*SPZ after immunization with radiation-attenuated *Py*SPZ and *Pb*SPZ

Immunization	SPZs in IFAT	IFAT titre
Irradiated *Py*SPZ	*P. yoelii*	2048
Irradiated *Py*SPZ	*P. berghei*	< 8
Irradiated *Pb*SPZ	*P. berghei*	4096
Irradiated *Pb*SPZ	*P. yoelii*	< 8

Mice were immunized i.v. with five doses of radiation-attenuated *Py*SPZ or *Pb*SPZ. Two weeks after the last immunization, just prior to challenge with infective SPZs, pooled sera were tested for antibodies to air-dried *Py*SPZ or *Pb*SPZ by IFAT.

### Cross-protection in BALB/c mice

Two weeks after the last immunization with radiation-attenuated SPZs, mice were challenged by i.v. injection of infective *Py*SPZ or *Pb*SPZ. In two challenge experiments, the number of *Py*SPZ and *Pb*SPZ used for challenge was 5 × 10^3^ and 10 × 10^3^, respectively. In all challenges, 100% of naive mice became infected. In these two challenges, protection against challenge with *Py*SPZ or *Pb*SPZ was 100% in groups that received the same parasite species for immunization and challenge (homologous immunization and challenge) (13 out of 13 mice protected for both parasites) (Table 1). However, BALB/c mice immunized with radiation-attenuated SPZs of either parasite strain showed different levels of cross-protection against the heterologous parasite species challenge. In two experiments, the level of cross-protection obtained when mice immunized with attenuated *Pb*SPZs were challenged with *Py*SPZs was greater (12 out of 13 mice protected, 92·3%) than when mice immunized with attenuated *Py*SPZs were challenged with *Pb*SPZ (7 out of 13 mice protected, 54%); *P =* 0·073, Fisher's exact test, two-sided. This difference occurred despite significantly higher infectivity of the *Py*SPZ (mean ID_50_ = 10·6) vs. *Pb*SPZ (mean ID_50_ = 571·1); *P* = 0·0047, independent samples *t*-test.

### Mechanisms of cross-protection in BALB/c mice

In BALB/c mice, CD8^+^ T cells have been shown to be responsible for the immunity against *Py*SPZ after immunization with radiation-attenuated *Py*SPZ (8–10), while protection induced in BALB/c mice after immunization with irradiated *Pb*SPZ is independent of CD8^+^ T cells (11). To determine whether the same immune mechanisms were responsible for the cross-protection between *P. yoelii*and *P. berghei*, we injected immunized mice with an anti-CD8 mAb and then challenged the mice with infective SPZs. The FACS analysis confirmed that injection of the mAb resulted in depletion of more than 99% of the CD8^+^ T cells (data not shown). Depletion of CD8^+^ T cells in BALB/c mice immunized with attenuated *Py*SPZ eliminated protection against challenge with homologous (*P. yoelii*) and heterologous (*P. berghei*) SPZs (Table 2). In mice immunized with attenuated *Pb*SPZs, treatment with anti-CD8 antibodies had no effect on challenge with homologous (*P. berghei*) SPZs, but it eliminated protection against heterologous (*P. yoelii*) SPZs (Table 3).

**Table 3:** Protective immunity in BALB/c mice immunized with irradiated *Pb*SPZs with and without CD8^+^ T cell depletion

			Number protected/number challenged		
Immunization	T cell depletion	Challenge SPZs^a^	Experiment 1	Experiment 2	Total (% protected)
Irradiated *Pb*SPZ	Control	*P. berghei*	ND	5/5	5/5 (100)
Irradiated *Pb*SPZ	CD8^+^	*P. berghei*	ND	5/5	5/5 (100)
Irradiated *Pb*SPZ	Control	*P. yoelii*	7/7	3/5	10/12 (83)
Irradiated *Pb*SPZ	CD8^+^	*P. yoelii*	0/6	0/6	0/12 (0)
Naive	−	*P. berghei*	ND	0/5	0/5 (0)
Naive	−	*P. yoelii*	0/6	0/6	0/12 (0)

Groups of BALB/c mice were immunized with five doses of radiation-attenuated *Pb*SPZ. Two weeks after the last immunization, mice were injected with anti-CD8 mAbs or control antibody prior to challenge with infective SPZs. The challenge dose for *P. berghei* was 4 × 10^3^ infective SPZs, and for *P. yoelii* was 1 × 10^3^ SPZs.

a The ID_50_ for *P. yoelii* used in Experiment 1 was 17·9 SPZs (95% CI 7·6−42·2), and the number of *P. yoelii* ID_50_ units contained in the 1 × 10^3^ SPZs used for this challenge was 55·9 (95% CI 23·7−132·0). ID_50_ units for the *P. yoelii* used in Experiment 2 and the *P. berghei* challenge could not be determined (see Materials and Methods).

ND, not done.

**Table 2:** Protective immunity in BALB/c mice immunized with radiation-attenuated *Py*SPZ with and without CD8^+^ T cell depletion

Immunization	T cell depletion	Challenge SPZs^a^	Number protected/ number challenged (% protected)
Irradiated *Py*SPZ	Control	*P. yoelii*	6/6 (100)
Irradiated *Py*SPZ	CD8^+^	*P. yoelii*	0/6 (0)
Irradiated *Py*SPZ	Control	*P. berghei*	5/6 (83)
Irradiated *Py*SPZ	CD8^+^	*P. berghei*	0/7 (0)
Naive	−	*P. yoelii*	0/6 (0)
Naive	−	*P. berghei*	0/6 (0)

Groups of BALB/c mice were immunized with five doses of radiation-attenuated *Py*SPZ. Two weeks after the last immunization, mice were injected with anti-CD8 mAbs or a control antibody prior to challenge with infective SPZs. The challenge dose for *P. berghei* was 4 × 10^3^ SPZs, and for *P. yoelii* was 10^3^ SPZs.

a The ID_50_ for *P. yoelii*was 13·6 SPZs (95% CI 5·4–34·0), and the number of *P. yoelii* ID_50_ units contained in the 1 × 10^3^ SPZs used for the challenge was 73·7 (95% CI 29·4−185·1). The ID_50_ units for the *P. berghei* challenge could not be determined (see Materials and Methods).

Since CD8^+^ T cell depletion did not affect protective efficacy in BALB/c mice immunized with radiation-attenuated *Pb*SPZ and challenged with *Pb*SPZs (Table 3), we assessed the effect of CD4^+^ T cell depletion in these mice (Table 5). Depletion of CD4^+^ T cells had no effect on protection (Table 5), but depletion of both CD4^+^ and CD8^+^ T cells had a modest effect on protective efficacy (Table 5). CD4^+^ T cell depletion reduced, but did not eliminate protection against challenge with *Py*SPZ.

**Table 5:** Protective immunity in BALB/c mice immunized with radiation-attenuated *Pb*SPZ with and without CD8^+^ and/or CD4^+^ T cell depletion

			Number protected/number challenged		
Immunization	T cell depletion	Challenge SPZs	Experiment 1	Experiment 2	Total (% protected)
Irradiated *Pb*SPZ	Control	*P. berghei*	7/7	7/7	14/14 (100)
Irradiated *Pb*SPZ	CD4^+^	*P. berghei*	7/7	ND	7/7 (100)
Irradiated *Pb*SPZ	CD4^+^ and CD8^+^	*P. berghei*	5/7	4/7	9/14 (64)
Irradiated *Pb*SPZ	Control	*P. yoelii*	4/7	5/7	9/14 (64)
Irradiated *Pb*SPZ	CD4^+^	*P. yoelii*	2/7	2/7	4/14 (29)
Naive	−	*P. berghei*	0/7	0/7	0/14 (0)
Naive	−	*P. yoelii*	0/7	0/7	0/14 (0)

Groups of BALB/c mice were immunized with five doses of radiation-attenuated *Pb*SPZ and depleted of either T cells expressing CD4^+^ or T cells expressing CD4^+^ and CD8^+^ markers, and challenged with 7 × 10^3^*Pb*SPZ or 1 × 10^3^ *Py*SPZ.

### Cross-protection between the two parasite species in C57BL/10 mice

In BALB/c mice immunized with radiation-attenuated *Pb*SPZ, CD8^+^ T cells are not required for protection against *Pb*SPZ challenge (11,12). However, in A/J (H-2a) mice immunized with radiation-attenuated *Pb*SPZ, CD8^+^ T cells are required for protection (12,13). Since the mechanism of protection against *P. berghei* varied among different mouse species, we wanted to determine if cross-protection extended to another mouse strain. We immunized C57BL/10 (H-2b) mice with irradiated *P. berghei* to determine the mechanism of protection induced in this mouse strain against homologous challenge and also to determine if mice immunized with irradiated *P. berghei* showed cross-protection against *Py*SPZ. In this mouse strain, C57BL/10 (H-2b), protection against the homologous *P. berghei* challenge was dependent on CD8^+^ T cells (Table 6). Furthermore, C57BL/10 (H-2b) mice immunized with irradiated *P. berghei* did not show cross-protection against *Py*SPZ (Table 6).

**Table 6:** Protective immunity in C57BL/10 mice immunized with radiation attenuated *Pb*SPZ

Immunization	T cell depletion	Challenge SPZs	Number protected/ number challenged (% protected)
*Experiment 1*			
Irradiated *Pb*SPZ	None	*P. berghei*	9/9 (100)
Irradiated *Pb*SPZ	None	*P. yoelii*	1/9 (11)
Naive	−	*P. berghei*	0/8 (0)
Naive	−	*P. yoelii*	1/9 (11)
*Experiment 2*			
Irradiated *Pb*SPZ	Control	*P. berghei*	5/5 (100)
Irradiated *Pb*SPZ	CD8^+^	*P. berghei*	0/5 (0)
Naive	−	*P. berghei*	0/5 (0)

Groups of C57BL/10 mice were immunized with five doses of irradiated *Pb*SPZ and challenged with either 5 × 10^3^*Pb*SPZ or *Py*SPZ (Experiment 1). In a second experiment (Experiment 2), C57BL/10 mice similarly immunized were depleted of their CD8^+^ T cell subpopulation and challenged with *Pb*SPZ.

## DISCUSSION

There were several major new findings in these studies. There was significant cross-protection between *P. yoelii* and *P. berghei* in BALB/c mice (Tables 1–3 and 5), antibodies against SPZs did not play a role in the cross-protection and the immune mechanisms required for protection against homologous challenge, but not heterologous challenge, in BALB/c mice were apparently different for *P. yoelii* (Table 2) and *P. berghei* (Tables 3 and 5).

The potentially most important finding was the cross-protection. One-hundred percent of mice were protected against homologous challenge. Thirty-one of 39 (79%) BALB/c mice immunized with attenuated *Pb*SPZ were protected against challenge with *Py*SPZ, and 12 of 19 (63%) BALB/c mice immunized with attenuated *Py*SPZ were protected against challenge with *Pb*SPZ (Tables 1–3 and 5).

Mice immunized with *Py*SPZ did not produce antibodies that recognized *Pb*SPZ, and mice immunized with *Pb*SPZ did not produce antibodies that recognized *Py*SPZ (Table 4). Thus, antibodies against SPZs could not have been responsible for the protection against heterologous challenge.

The protection against heterologous challenge in mice immunized with either radiation-attenuated *Py*SPZ or *Pb*SPZ was dependent on CD8^+^ T cells (Tables 2 and 3). When mice were immunized with radiation-attenuated *Py*SPZ and challenged with nonirradiated *Pb*SPZ, or immunized with radiation-attenuated *Pb*SPZ and challenged with nonirradiated *Py*SPZ, the protective immunity was eliminated by treatment of the mice before challenge with an antibody to CD8^+^ T cells. These data demonstrated that CD8^+^ T cells were required for the cross-protection. The requirement for T cells to provide protection against heterologous parasites was consistent with the finding that these mice made no antibodies against the heterologous SPZs (Table 4).

In BALB/c mice immunized with radiation-attenuated *Py*SPZ, protection against challenge with *P. yoelii* was dependent on CD8^+^ T cells (Table 2). However, in BALB/c mice immunized with radiation-attenuated *Pb*SPZ, protection against *P. berghei* was not eliminated by depletion of either CD8^+^ (Table 3) or CD4^+^ T cells (Table 5) and only modestly reduced by depletion of both (Table 5). The finding of lack of dependence on CD8^+^ T cells in BALB/c mice immunized by i.v. injection of irradiated *Pb*SPZ model was first reported more than 16 years ago (11) Interestingly this did not occur in mice immunized by the bite of irradiated, *Pb*SPZ-infected mosquitoes (14). Regardless, the other results have not been previously reported. There are several possible explanations for why T cell depletion did not reduce protection against *P. berghei* in mice immunized with irradiated *Pb*SPZ. One possibility is that non-T cell immunity is more effective against *Pb*SPZ infection than it is against *P. yoelii*. This could be because the infectivity (ID_50_) of *Pb*SPZ is lower than that of *Py*SPZ (Table 1 and see below), and the parasites are easier to eliminate by non-T cell mechanisms because fewer of them are infective. This differential infectivity could explain why, in contrast to *Pb*SPZ, the protection against *Py*SPZ in these same mice was eliminated by depletion of CD8^+^ T cells. Another explanation for why T cell depletion of mice immunized with irradiated *Pb*SPZ did not eliminate protection is that the effect of *in vivo* T cell depletion on effector T cells could have been less in mice immunized with irradiated *Pb*SPZ as compared to after immunization with irradiated *Py*SPZ. Although the efficiency of *in vivo* depletion of total splenic CD8^+^ and CD4^+^ T cells was 99% in these experiments, we did not measure antigen-specific T cell populations responding to *P. berghei* or *P. yoelii* antigens after depletion. Thus, there remains a possibility that sufficient effector cells remained in the residual 1% of undepleted total T cells to protect mice against *Pb*SPZ infection, but not *P. yoelii* infection. There is a region, amino acids 281–289 on the *P. yoelii* circumsporozoite protein (*Py*CSP) (SYVPSAEQI) and *P. berghei*circumsporozoite protein (*Pb*CSP) (SYIPSAEKI), which contains a protective cytotoxic T lymphocytes (CTL) epitope (15). Preliminary studies by one of us (W. Weiss, unpublished data) indicate that the precursor frequency of CD8^+^ CTLs responding to the specific epitopes were 10-fold higher in BALB/c mice after immunization with irradiated *Pb*SPZ than with *Py*SPZ. Thus, while *in vivo* depletion removed 99% of all CD8^+^ T cells, it is possible that it removed 10-fold less CD8^+^ T effector cells against this protective CTL epitope in mice immunized with irradiated *Pb*SPZ as compared to irradiated *Py*SPZ. Thus, it is possible that T cells were responsible for protection of BALB/c mice immunized with irradiated *Pb*SPZ, but that our methods of *in vivo* depletion were inadequate to demonstrate this. This line of reasoning does not explain why in these same mice T cell depletion eliminated protection against challenge with *Py*SPZ.

In mice immunized with irradiated *Pb*SPZ and challenged with *Py*SPZ, CD4^+^ T cell depletion modestly reduced protection (Table 5), indicating that CD4^+^ T cells play a role in this heterologous protection. Interestingly, this effect was not seen with homologous challenge, but depletion of both CD8^+^ and CD4^+^ T cells reduced protection against homologous (*P. berghei*) challenge, again showing that CD4^+^ T cells do play some role in protection. This finding also raises the possibility of the complementary effects of CD8+ and CD4^+^ T cells working together. Nevertheless, our findings and more than 20 years of research on mice immunized with irradiated SPZ have demonstrated that CD8^+^ T cells are the major effector arm of the immune system, but that CD4^+^ T cells also play a role, albeit minor role, and that in some cases, antibodies can be the major effector immune response (16).

Despite the excellent cross-protection in BALB/c mice, there was no cross-protection in C57BL/10 mice. It is possible that the immune response induced with the irradiated *Pb*SPZ was not strong enough to overcome the highly infectious *Py*SPZ challenge in the C57BL/10 mice. The high susceptibility of C57BL/10 mice to *Py*SPZ may also explain why in some experiments not all mice immunized with irradiated *Py*SPZ become protected after challenge with *Py*SPZ (12,17).

It is generally thought that optimally radiation-attenuated SPZs pass through the bloodstream to the liver, invade hepatocytes and partially develop expressing proteins not expressed in hepatocytes. Many malariologists believe the CD8^+^ T cell-dependent protective immunity elicited by immunization with radiation-attenuated SPZs is primarily directed against these ‘new’ proteins first expressed in hepatocytes (10,16,17) This view has been based on a report that ‘over-irradiated’ SPZs do not provide protection even though they invade hepatoctyes (18), and killed SPZs do not provide high level of protection (19). Thus, despite the fact that immunization with *Py*CSP and *Pb*CSP (11,20–24) based vaccines elicit CD8^+^ T cell-dependent protection against homologous challenge, and transfer of an anti-*Py*CSP T cell clone protects against *P. yoelii* and *P. berghei* challenge (15,25), it is thought that these SPZ-derived proteins are not adequate for the high level of protective immunity seen after immunization with radiation-attenuated SPZs. These conclusions are supported by a recent study with mice transgenic for the *Py*CSP, and therefore tolerant to the *Py*CSP (26,27). When these mice received two doses of irradiated *Py*SPZ, there was only minimal protection, indicating that *Py*CSP was important in protective immunity. However, when these mice received a full immunizing regimen of three doses of irradiated *Py*SPZ, they were fully protected against challenge with infectious *Py*SPZ, indicating that in this model system, immune responses against the *Py*CSP were not required for protection. While the data reported herein do not indicate what antigens are responsible for the protection, the findings that a *Py*CSP T cell clone was protective against challenge with the heterologous *Pb*SPZ (15) possibly through the mediation of antigen-specific induction of IFNγ is significant. The availability of the genomic sequences of *P. yoelii* (28) and *P. berghei* (29), and gene expression and proteomic analyses should facilitate the discovery of *other* antigens involved.

The data presented in this paper re-emphasize the differences in infectivity of *Py*SPZ and *Pb*SPZ (30–32). The mean ID_50_ of *P. yoelii*was 10·6 (95% CI 6·1–18·3) while that of *P. berghei* was 571·1 (249·9–1304·8), indicating that far less SPZs are needed to produce a *P. yoelii* infection.

The finding of cross-protection in mice between *P. yoelii* and *P. berghei* after immunization with radiation-attenuated SPZs is provocative, but cannot be used to predict what will occur in humans immunized with radiation-attenuated SPZs. However, when the radiation-attenuated *Pf*SPZ vaccine is developed and tested (33), it will be important to determine if immunization with this vaccine protects against *P. vivax*. The data presented herein suggest that cross-protection could occur. Comparative analyses of the genomes of *P. falciparum, P. vivax, P. yoelii*and *P. berghei*may provide more insight into the likelihood of cross-protection. However, there will be no substitute for carefully executed clinical trials to determine if there is cross-protection.
